# Analysis of the clinical utility of p16/Ki-67 dual staining in screening cervical lesions among women positive for high-risk HPV

**DOI:** 10.1097/MD.0000000000050102

**Published:** 2026-08-21

**Authors:** Qingsong Zhang, Yunpeng Lin, Leilei Bao, Qiuyang Huang

**Affiliations:** a Department of Clinical Laboratory, Cangnan Hospital of Traditional Chinese Medicine, Cangnan County, Wenzhou, Zhejiang Province, China; b Department of Gynecology, Cangnan Hospital of Traditional Chinese Medicine, Cangnan County, Wenzhou, Zhejiang Province, China.

**Keywords:** cervical cancer, cervical intraepithelial neoplasia, dual staining, high-risk human papillomavirus, p16/Ki-67 dual staining, screening, triage strategy

## Abstract

Cervical cancer is closely associated with persistent infection by high-risk human papillomavirus (hrHPV). Although HPV testing offers high sensitivity, its limited specificity may result in excessive colposcopy referrals, and the triage performance of traditional liquid-based cytology (TCT) remains suboptimal among hrHPV-positive women. As a biomarker reflecting HPV-mediated cell-cycle dysregulation and aberrant proliferation, p16/Ki-67 dual staining has shown potential advantages in identifying clinically significant high-grade cervical lesions. This study aimed to evaluate the clinical utility of p16/Ki-67 dual staining in screening cervical lesions among hrHPV-positive women and to explore its role in optimizing referral strategies. Retrospectively, this single-center cohort study included 100 consecutive hrHPV-positive women who attended our clinic between October 2024 and October 2025. All participants underwent hrHPV genotyping, TCT, and p16/Ki-67 dual staining during the same visit, followed by colposcopy and histopathological assessment. Histology served as the reference standard. We evaluated the diagnostic performance of p16/Ki-67 for cervical intraepithelial neoplasia 2+ (CIN2+) lesions and compared it with TCT, HPV genotyping, and a combined triage model (hrHPV + TCT + p16/Ki-67). In addition, we simulated the screening efficiency of different colposcopy referral strategies. Among the 100 hrHPV-positive women included, 35 (35.0%) were diagnosed with CIN2+. The prevalence of HPV16 infection and the positivity rate of p16/Ki-67 dual staining were significantly higher in the CIN2+ group than in the non-CIN2+ group (*P* < .01). For CIN2+ detection, p16/Ki-67 dual staining demonstrated a sensitivity of 88.6%, a specificity of 78.5%, and a negative predictive value of 93.0%. Dual-stain positivity increased progressively with cytological severity and provided meaningful triage value across atypical squamous cells of undetermined significance, low-grade squamous intraepithelial lesion, and high-grade squamous intraepithelial lesion categories. The combined triage model yielded the best diagnostic performance, with an area under the curve of 0.91. Using “dual-stain positivity or TCT ≥ atypical squamous cells of undetermined significance” as the referral threshold substantially reduced the colposcopy referral rate while maintaining a high CIN2+ detection rate. p16/Ki-67 dual staining provides high diagnostic accuracy and strong negative predictive capability for CIN2+ lesions among hrHPV-positive women. Integrating p16/Ki-67 dual staining with TCT and HPV genotyping can markedly improve screening precision and optimize colposcopy referral strategies, thereby reducing unnecessary invasive procedures and demonstrating promising clinical applicability.

## 1. Introduction

Cervical cancer remains one of the most prevalent malignancies affecting women worldwide, with >600,000 new cases reported annually. Its development is strongly driven by persistent infection with high-risk human papillomavirus (hrHPV).^[1]^ Among the oncogenic HPV types, HPV16 and HPV18 account for the majority of cervical intraepithelial neoplasia (CIN) and invasive cervical cancers, with HPV16 demonstrating the greatest carcinogenic potential and conferring a markedly elevated risk of CIN2/3 and malignancy.^[2,3]^ Given the predictable progression pattern of CIN, cervical cancer is considered one of the most preventable cancers, and early detection of clinically relevant high-grade lesions is essential for reducing disease burden.^[4]^

Conventional screening modalities – liquid-based cytology (thinprep cytologic test [TCT]/Pap) and HPV DNA testing – each present inherent limitations. Cytology offers high specificity but insufficient sensitivity, whereas HPV testing, despite its strong sensitivity, lacks the ability to discriminate lesions with true malignant potential, leading to excessive colposcopy referrals and inefficient use of medical resources.^[5–7]^ Identifying more effective triage tools for hrHPV-positive women has therefore become a priority in contemporary screening strategies.

The biomarker p16 INK4a, a cyclin-dependent kinase inhibitor, is overexpressed when the HPV E7 oncoprotein disrupts the Rb pathway. Ki-67 reflects active cellular proliferation. These markers should not co-express in normal epithelial cells; their simultaneous positivity within the same cell indicates HPV-related deregulated proliferation, which is strongly associated with CIN2+ lesions.^[8,9]^ This biological signature provides the rationale for p16/Ki-67 dual staining as a molecular method for detecting high-grade cervical disease.

An increasing body of evidence demonstrates that p16/Ki-67 dual staining outperforms cytology in sensitivity and negative predictive value among HPV-positive women, particularly in triaging those with atypical squamous cells of undetermined significance (ASC-US) or low-grade squamous intraepithelial lesion (LSIL), where cytology alone is insufficient. Its application can reduce unnecessary colposcopy and enhance the detection of CIN2+ lesions.^[10–12]^ Large prospective studies further report that the diagnostic sensitivity of dual staining for CIN2+ may exceed 90%, with especially strong performance in women infected with HPV16/18.^[13]^

Given the complementary predictive value of HPV genotyping, cytology, and p16/Ki-67 dual staining, recent guidelines increasingly advocate for risk-based, multi-indicator screening models to improve accuracy and cost-effectiveness.^[14]^ Moreover, dual staining has shown diagnostic utility even in cytology-negative but HPV-positive women, offering an opportunity to recognize otherwise occult high-grade lesions.^[15]^ Collectively, current evidence highlights the significant potential of p16/Ki-67 dual staining in the triage of hrHPV-positive women, underscoring the need for further validation in real-world clinical populations and for refining optimized triage algorithms.

## 2. Methods

### 2.1. Study design, study period, and data sources

This retrospective, single-center cohort study was approved by the Ethics Committee of Cangnan Hospital of Traditional Chinese Medicine and was conducted to evaluate the clinical performance of p16/Ki-67 dual staining in screening cervical lesions among women positive for hrHPV. The study period extended from October 1, 2024 to October 31, 2025. Data were obtained from the gynecology outpatient department, pathology archive, and laboratory information system. A total of 100 consecutive eligible hrHPV-positive women were included; all underwent hrHPV genotyping, liquid-based cytology (TCT), and p16/Ki-67 dual staining during the same clinical encounter, followed by colposcopy and histopathological assessment according to clinical indications. The study complied with the Declaration of Helsinki. Because this was a retrospective study using de-identified data, the requirement for informed consent was waived.

Sample size was determined by the number of consecutive patients meeting all eligibility criteria during the prespecified study period rather than by an a priori hypothesis-driven calculation. This real-world exploratory cohort was intended to generate preliminary estimates of diagnostic performance and referral efficiency. With 35 CIN2+ cases and 65 non-CIN2+ cases, the study provides clinically informative point estimates, but the precision of subgroup analyses is limited; therefore, all findings should be interpreted as exploratory and require confirmation in larger prospective multicenter cohorts.

### 2.2. Inclusion and exclusion criteria

Inclusion criteria were:

1. Female patients aged 21 to 65 years;
2. Positivity for HPV16, HPV18, or other hrHPV genotypes;
3. Completion of both TCT and p16/Ki-67 dual staining during the study period;
4. Availability of complete clinical information;
5. Absence of prior treatment for cervical lesions.

Exclusion criteria were:

1. History of cervical cancer or CIN2+ treatment;
2. Pregnancy or postpartum period < 6 months;
3. Unreadable cytology due to acute vaginitis or cervicitis;
4. Substandard specimen quality;
5. Missing key clinical data.

### 2.3. HPV genotyping and cytological evaluation

hrHPV genotyping was performed using nucleic acid amplification combined with gene-chip hybridization, detecting HPV16, HPV18, and other high-risk genotypes. Liquid-based cytology followed standardized thin-layer preparation procedures, and results were classified according to the Bethesda System, including negative for intraepithelial lesion or malignancy, ASC-US, atypical squamous cells, cannot exclude high-grade squamous intraepithelial lesion, LSIL, and high-grade squamous intraepithelial lesion (HSIL). Two cytopathologists independently interpreted the slides, with discrepancies adjudicated by a senior reviewer.

### 2.4. p16/Ki-67 dual staining procedure and interpretation

p16/Ki-67 dual staining was performed using a commercial ready-to-use immunocytochemistry kit on an automated staining platform. A specimen was considered positive when the same epithelial cell exhibited nuclear Ki-67 positivity together with cytoplasmic and/or nuclear p16 overexpression. All slides were independently reviewed by 2 experienced pathologists to ensure consistency.

### 2.5. Colposcopic examination and histopathological assessment

Colposcopy was conducted based on screening results, documenting acetowhite changes, iodine staining patterns, and abnormal lesion features. Directed biopsies were obtained from suspicious areas. Histological diagnoses were rendered by 2 senior pathologists on hematoxylin–eosin–stained sections. Pathology was categorized as normal, CIN1, CIN2, CIN3, or invasive carcinoma, with CIN2+ defined as high-grade disease.

### 2.6. Outcome measures

This study assessed the following parameters:

1. Diagnostic performance of p16/Ki-67 dual staining: sensitivity, specificity, positive predictive value (PPV), and negative predictive value (NPV) for detecting CIN2+, evaluating its role as a triage tool for hrHPV-positive women.
2. Joint screening performance with TCT and HPV genotyping: CIN2+ detection across different cytology subgroups (ASC-US, LSIL) and comparison of dual-stain positivity between HPV16/18 and non-16/18 hrHPV genotypes.
3. Risk-based triage modeling and referral strategy evaluation: construction of a combined hrHPV + TCT + p16/Ki-67 model; calculation of the area under the receiver operating characteristic curve (area under the curve [AUC]); and assessment of referral thresholds (e.g., dual-stain positivity) to balance colposcopy reduction with CIN2+ detection efficiency.

### 2.7. Data management and quality control

All raw data were collected by trained research staff and entered using a dual-entry approach; discrepancies were resolved by a 3rd investigator. To reduce review bias, cytopathologists and dual-stain reviewers were blinded to histopathological outcomes, and histopathologists were blinded to cytology and p16/Ki-67 results when rendering the reference diagnosis. Disagreements were adjudicated by a senior reviewer. Nevertheless, several potential sources of bias remained: selection bias because only women completing the required tests and histological verification were included; verification/work-up bias because biopsy procedures were guided by clinical and colposcopic findings; information bias arising from retrospective records and interobserver interpretation; and spectrum bias due to the single-center referral population. Consecutive enrollment, standardized testing procedures, predefined criteria, independent readings, and double data entry were used to mitigate these risks, but residual bias cannot be excluded.

### 2.8. Statistical analysis

Statistical analyses were conducted using SPSS version 22. Continuous variables were assessed for normality using the Shapiro–Wilk test and inspection of *Q–Q* plots. Normally distributed variables were summarized as mean ± standard deviation and compared using the independent-samples t test; non-normally distributed variables were summarized as median (interquartile range) and compared using the Mann–Whitney *U* test. Categorical variables were reported as frequencies and percentages and compared using the χ^2^ test or Fisher exact test, as appropriate. Using histopathology as the reference standard, sensitivity, specificity, PPV, NPV, likelihood ratios, and receiver operating characteristic curves with corresponding AUC values and 95% confidence intervals were calculated. A 2-sided *P* < .05 was considered statistically significant. Because this was an exploratory study with a modest sample size, no multiplicity adjustment was applied, and subgroup findings were interpreted cautiously.

## 3. Results

### 3.1. Baseline characteristics

Among the 100 hrHPV-positive women included in this study, 35 were diagnosed with CIN2+ and 65 were classified as non-CIN2+. Normality of age and body mass index was assessed using the Shapiro–Wilk test and *Q–Q* plots. Both variables were approximately normally distributed and were therefore summarized as mean ± standard deviation and compared using independent-samples *t* tests. Age and body mass index did not differ significantly between groups (*P* > .05). The distribution of HPV genotypes showed a higher prevalence of HPV16 in the CIN2+ group (48.6% vs 21.5%, χ^2^ = 7.98, *P* = .005), whereas non-16/18 hrHPV types were more common in the non-CIN2+ group (69.2% vs 37.1%, *P* = .002) (Table 1). Cytology also differed between groups: 85.7% of women with CIN2+ had TCT results of ASC-US or higher, compared with 43.1% in the non-CIN2+ group (*P* < .001). HSIL was more frequent in the CIN2+ group (42.9% vs 6.2%, *P* < .001). Dual-stain positivity was 88.6% in the CIN2+ group and 21.5% in the non-CIN2+ group (*P* < .001).

**Table 1:** Comparison of baseline characteristics.

Variable	CIN2+ group (n = 35)	Non-CIN2+ group (n = 65)	*t*/χ^2^	*P* value
Age (yr, mean ± SD)	38.7 ± 8.4	40.2 ± 9.1	0.82	.415
BMI (kg/m^2^)	23.4 ± 2.8	22.9 ± 3.1	0.81	.42
History of smoking (%)	9 (25.7%)	8 (12.3%)	2.87	.09
Multiparity (≥2 births) (%)	18 (51.4%)	22 (33.8%)	2.8	.094
HPV16 positive (%)	17 (48.6%)	14 (21.5%)	7.98	.005
HPV18 positive (%)	5 (14.3%)	6 (9.2%)	0.57	.45
Other hrHPV (%)	13 (37.1%)	45 (69.2%)	9.66	.002
TCT ≥ ASC-US (%)	30 (85.7%)	28 (43.1%)	18.94	<.001
TCT HSIL (%)	15 (42.9%)	4 (6.2%)	22.09	<.001
p16/Ki-67 dual staining positive (%)	31 (88.6%)	14 (21.5%)	42.59	<.001

ASC-US = atypical squamous cells of undetermined significance, BMI = body mass index, CIN = cervical intraepithelial neoplasia, HPV = human papillomavirus, hrHPV = high-risk human papillomavirus, HSIL = high-grade squamous intraepithelial lesion, SD = standard deviation, TCT = thinprep cytologic test.

### 3.2. Relationship between HPV genotype and p16/Ki-67 dual-stain positivity

When stratified by HPV genotype (HPV16, HPV18, dual 16/18 infection, and other hrHPV types), significant differences in dual-stain positivity were observed (Table 2). HPV16-positive women demonstrated the highest positivity rate (77.4%), exceeding that observed in HPV18 (54.5%) and other hrHPV infections (20.8%). Statistical analysis confirmed a robust correlation between HPV16 and dual-stain positivity (χ^2^ = 21.56, *P* < .001). These findings reflect the close alignment between HPV16 infection and alterations in cell-cycle regulation marked by co-expression of p16 and Ki-67. In contrast, fewer than one-fifth of infections with other hrHPV types exhibited dual positivity, indicating a comparatively lower oncogenic potential. Such stratification highlights the utility of genotype-based risk refinement in screening practice.

**Table 2:** Association between HPV genotype and p16/Ki-67 dual-stain positivity.

HPV genotype	Total cases	Dual-stain positive n (%)	Dual-stain negative n (%)	χ^2^ for positivity difference	*P* value
HPV16	31	24 (77.4%)	7 (22.6%)	21.56	<.001
HPV18	11	6 (54.5%)	5 (45.5%)	–	–
HPV16/18 double positive	5	4 (80.0%)	1 (20.0%)	–	–
Other hrHPV (12 types)	53	11 (20.8%)	42 (79.2%)	–	–
Total	100	45 (45.0%)	55 (55.0%)	–	–

HPV = human papillomavirus, hrHPV = high-risk human papillomavirus.

### 3.3. TCT category and p16/Ki-67 dual-stain positivity

Dual-stain positivity increased progressively with cytological severity (Table 3). The positivity rate was 13.3% in negative for intraepithelial lesion or malignancy, rising to 42.9% in ASC-US, 59.1% in LSIL, and reaching 80.0% among HSIL cases. The predictive strength of dual staining for CIN2+ also escalated across TCT categories; sensitivities in LSIL and HSIL were 88.9% and 94.4%, respectively. These results emphasize the key role of dual staining in triaging women with minor cytologic abnormalities (ASC-US/LSIL), helping identify those requiring timely colposcopy.

**Table 3:** TCT classification and p16/Ki-67 dual-stain positivity.

TCT result	n	Dual-stain positive n (%)	Dual-stain negative n (%)	CIN2+ cases	Sensitivity of dual staining for CIN2+ within category
NILM	30	4 (13.3%)	26 (86.7%)	2	50.00%
ASC-US	28	12 (42.9%)	16 (57.1%)	6	83.30%
LSIL	22	13 (59.1%)	9 (40.9%)	9	88.90%
HSIL	20	16 (80.0%)	4 (20.0%)	18	94.40%

ASC-US = atypical squamous cells of undetermined significance, CIN = cervical intraepithelial neoplasia, HSIL = high-grade squamous intraepithelial lesion, LSIL = low-grade squamous intraepithelial lesion, NILM = negative for intraepithelial lesion or malignancy, TCT = thinprep cytologic test.

### 3.4. Colposcopic findings and histopathologic outcomes

Colposcopic impressions aligned well with final histopathologic diagnoses (Table 4). Acetowhite lesions and iodine-negative areas were noted in 45 and 38 patients, respectively, and approximately half of these cases were confirmed as CIN2+. Of particular significance, 83.3% of patients with atypical vascular patterns had CIN2+, including 2 cases of invasive cancer, underscoring abnormal vasculature as a strong indicator of high-grade or invasive pathology. Conversely, none of the 17 women with “no remarkable colposcopic abnormality” were diagnosed with CIN2+, reaffirming colposcopy as an effective lesion-localization tool. These findings also suggest potential added value when combining dual staining with colposcopic assessment.

**Table 4:** Correspondence between colposcopic findings and histopathology.

Colposcopic impression	Total	Normal	CIN1	CIN2	CIN3	Cancer	CIN2+ proportion (%)
Suspicious acetowhite epithelium	45	4	18	10	11	2	51.10%
Iodine-negative or weak-staining areas	38	6	12	8	10	2	52.60%
Suspicious vascular abnormalities	12	0	2	4	4	2	83.30%
No obvious abnormality	17	14	3	0	0	0	0%

CIN = cervical intraepithelial neoplasia.

### 3.5. Diagnostic performance of p16/Ki-67 dual staining for CIN2+

Dual staining demonstrated useful diagnostic performance for CIN2+ in this cohort (Table 5). Sensitivity was 88.6%, specificity was 78.5%, and the negative predictive value was 93.0%. These findings indicate a relatively low residual probability of CIN2+ after a negative dual-stain result in the present sample, but they should not be interpreted as complete exclusion of high-grade disease. The positive likelihood ratio was 4.12 and the negative likelihood ratio was 0.15. The Youden index was 0.671. Collectively, these results support further evaluation of p16/Ki-67 dual staining as a triage method among hrHPV-positive women.

**Table 5:** Diagnostic performance of p16/Ki-67 dual staining for CIN2+.

Parameter	Value
Sensitivity	88.60%
Specificity	78.50%
Positive predictive value (PPV)	68.90%
Negative predictive value (NPV)	93.00%
Accuracy	81.00%
Positive likelihood ratio (LR+)	4.12
Negative likelihood ratio (LR−)	0.15
Youden index	0.671

CIN = cervical intraepithelial neoplasia.

### 3.6. Performance of the combined triage model

Comparative evaluation of HPV genotype, cytology, dual staining, and their combined model (Table 6) showed that the integrated hrHPV + TCT + p16/Ki-67 model yielded the highest diagnostic accuracy. Its AUC reached 0.91 (95% confidence interval: 0.85–0.97), outperforming any individual test or dual-test combination. Sensitivity and specificity were 94.3% and 82.3%, respectively, with PPV and NPV of 79.2% and 95.5%. In contrast, TCT ≥ ASC-US alone demonstrated good sensitivity (85.7%) but poor specificity (56.9%), potentially leading to excessive colposcopy referrals. These results highlight the combined model’s promise for enhancing overall screening efficiency and precision.

**Table 6:** Diagnostic performance of the triple-combination model (HPV + TCT + dual staining).

Screening strategy	AUC	95% CI	Sensitivity	Specificity	PPV	NPV	Interpretation
HPV16/18 alone	0.68	0.57–0.78	62.90%	69.20%	52.40%	77.80%	Genotype informative but insufficient for triage
TCT ≥ ASC-US	0.74	0.64–0.83	85.70%	56.90%	51.70%	88.00%	High sensitivity, low specificity
p16/Ki-67 dual staining	0.83	0.75–0.91	88.60%	78.50%	68.90%	93.00%	Best single-test performance
Combined model (HPV + TCT + dual stain)	0.91	0.85–0.97	94.30%	82.30%	79.20%	95.50%	Optimal strategy

AUC = area under the curve, CI = confidence interval, HPV = human papillomavirus, NPV = negative predictive value, PPV = positive predictive value, TCT = thinprep cytologic test.

### 3.7. Impact of different colposcopy referral strategies on screening efficiency

Simulation of various referral algorithms (Table 7) revealed major differences in clinical workload and yield. Universal referral of all hrHPV-positive women ensured complete CIN2+ detection but resulted in a 100% referral rate, which is impractical in real-world settings. Using dual-stain positivity as the sole referral criterion reduced the referral rate to 45% while maintaining an 88.6% detection rate for CIN2+, representing a highly efficient strategy. The proposed approach of “TCT ≥ ASC-US or dual-stain positivity” showed an optimal balance: referral rate 63%, CIN2+ detection rate 94.3%, and only 2 missed cases. These results indicate that incorporating dual staining into the triage of ASC-US/LSIL patients can markedly reduce unnecessary colposcopy while preserving high sensitivity.

**Table 7:** Efficiency analysis of different colposcopy referral strategies.

Referral strategy	No. referral	Referral rate	CIN2+ detection rate	PPV of colposcopy	CIN2+ missed	Notes
Refer all hrHPV-positive	100	100%	100%	35%	0	Most conservative
Refer for TCT ≥ ASC-US	48	48%	85.70%	62.50%	5	Low specificity
Refer for dual-stain positivity	45	45%	88.60%	68.90%	4	Highly efficient
HPV16/18 or TCT ≥ ASC-US	56	56%	91.40%	57.10%	3	Clinically practical
Dual-stain positive OR TCT ≥ ASC-US (recommended)	63	63%	94.30%	52.40%	2	Best balance of efficiency and detection
If TCT = ASC-US, use dual stain for triage	40	40%	82.90%	72.50%	6	Precision-focused

ASC-US = atypical squamous cells of undetermined significance, CIN = cervical intraepithelial neoplasia, hrHPV = high-risk human papillomavirus, OR = odds ratio, PPV = positive predictive value, TCT = thinprep cytologic test.

## 4. Discussion

This single-center retrospective cohort study evaluated the potential clinical utility of p16/Ki-67 dual staining for triaging hrHPV-positive women. In this selected cohort, dual staining showed a sensitivity of 88.6% and an NPV of 93.0% for CIN2+. These estimates suggest that dual staining may contribute to risk stratification, but they should be interpreted in the context of the retrospective design, modest sample size, relatively high CIN2+ prevalence, and histology-verification pathway. The results are broadly consistent with previous studies, while not establishing superiority or clinical effectiveness beyond this study setting.^[16,17]^

Previous studies have shown that the combined detection of p16 overexpression and Ki-67 indicates HPV-driven cell-cycle dysregulation and aberrant proliferation, enabling superior discrimination of clinically significant CIN2+ lesions compared with cytology alone.^[16–18]^ Wentzensen et al reported that dual staining yields higher sensitivity and NPV than conventional cytology among HPV-positive women, allowing reliable exclusion of short-term progression to high-grade disease.^[16]^ Our findings are consistent with this observation, as the likelihood of CIN2+ among dual-stain–negative individuals was extremely low, highlighting its value in determining safe follow-up intervals.

Our study also confirmed that HPV16 infection was associated with markedly higher dual-stain positivity compared with other hrHPV genotypes, a pattern well documented in molecular epidemiologic studies.^[19,20]^ The stronger oncogenic activity of HPV16 E6/E7 promotes p16 overexpression and uncontrolled cellular proliferation, resulting in high rates of dual-marker expression.^[19]^ Supporting this, Trimble et al showed that dual staining performed particularly well in detecting CIN3+ among HPV16-positive women.^[20]^ Thus, integrating genotype information with dual staining may facilitate refined risk stratification.

ASC-US and LSIL represent the most frequent but clinically challenging categories in cervical screening, often leading to unnecessary colposcopy referrals when cytology alone is used.^[21]^ Multiple studies have demonstrated that incorporating dual staining into the triage of women with minor cytologic abnormalities significantly improves CIN2+ detection while reducing unnecessary procedures.^[22,23]^ Ye et al further confirmed its real-world effectiveness, showing a reduced referral burden without compromising sensitivity.^[22]^ Our findings reinforce these conclusions, as dual staining showed robust predictive value in both ASC-US and LSIL subgroups.

Although colposcopy remains central to lesion localization and biopsy guidance, its interpretation relies heavily on examiner expertise and is subject to variability.^[24]^ In this study, dual-stain positivity showed strong concordance with suspicious colposcopic findings and with histologically confirmed CIN2+, particularly in cases with atypical vascular patterns – features often associated with high-grade or invasive disease. This observation is consistent with the work of Gothwal et al, who proposed dual staining as a valuable objective adjunct prior to colposcopy referral.^[25]^

Risk-based, multi-parameter screening models have gained prominence as strategies to optimize cervical cancer prevention.^[26]^ The combined model in our study – integrating hrHPV genotyping, cytology, and dual staining – achieved superior AUC, sensitivity, and specificity compared with single-test or dual-test approaches. Similar findings have been reported in European and North American cohorts, where combining molecular biomarkers with cytologic assessment improved detection accuracy and reduced the overall healthcare burden.^[27,28]^ The American Society for Colposcopy and Cervical Pathology management framework proposed by Egemen et al also emphasizes the need to incorporate molecular tests into risk-based decision algorithms.^[26]^

Balancing referral reduction with the avoidance of missed CIN2+ cases remains a key challenge in cervical screening. In our simulation, the rule “dual-stain positivity or TCT ≥ ASC-US” produced a referral rate of 63% and detected 94.3% of CIN2+ cases, with 2 CIN2+ cases missed. This trade-off may be clinically relevant, but the strategy was evaluated retrospectively in the same dataset used to construct the model and was not externally validated. Accordingly, the proposed threshold should be regarded as hypothesis-generating rather than ready for routine implementation. Prospective validation should assess safety, reproducibility, patient acceptability, resource use, and cost-effectiveness, including outcomes among women with negative dual staining.^[29,30]^

This study has several limitations. First, it was a retrospective analysis from a single hospital in Zhejiang Province, China. Local referral patterns, HPV genotype distribution, screening participation, laboratory procedures, and disease prevalence may differ from those in population-based programs, other regions of China, and other countries; therefore, the diagnostic values and simulated referral rates may not be directly generalizable. Second, the modest sample size, including only 35 CIN2+ cases, limited statistical precision and prevented robust age-, genotype-, and cytology-specific analyses. The sample was based on all consecutive eligible cases during the study period, without an a priori power calculation, so subgroup findings are exploratory. Third, inclusion required completion of TCT, dual staining, colposcopy, and histological assessment, which may have introduced selection and verification bias and may explain the relatively high prevalence of CIN2+. Fourth, retrospective data collection may be affected by incomplete documentation and uncontrolled confounding. Although independent blinded readings and standardized procedures were used, interobserver variability in cytology, dual-stain interpretation, colposcopy, and histopathology cannot be eliminated. Fifth, the combined model and referral strategies were developed and evaluated in the same cohort, creating a risk of overfitting and optimistic performance estimates; no internal resampling or external validation was performed. Sixth, follow-up was insufficient to determine the longitudinal safety of deferring colposcopy in dual-stain-negative women, and genotype-specific effects beyond HPV16/18 were not examined in detail. Future studies should use prospective multicenter designs, larger and more diverse population-based samples, standardized verification protocols, blinded central review, prespecified sample-size calculations, external validation, and longitudinal and cost-effectiveness analyses before clinical implementation.

## Author contributions

**Conceptualization:** Qingsong Zhang, Yunpeng Lin, Leilei Bao, Qiuyang Huang.

**Data curation:** Qingsong Zhang, Yunpeng Lin, Leilei Bao, Qiuyang Huang.

**Formal analysis:** Qingsong Zhang, Yunpeng Lin, Leilei Bao, Qiuyang Huang.

**Funding acquisition:** Qiuyang Huang.

**Investigation:** Qiuyang Huang.

**Writing – original draft:** Qiuyang Huang.

**Writing – review & editing:** Qiuyang Huang.
